# Effects of 1α,25 dihydroxyvitamin D3 and testosterone on miRNA and mRNA expression in LNCaP cells

**DOI:** 10.1186/1476-4598-10-58

**Published:** 2011-05-18

**Authors:** Wei-Lin W Wang, Namita Chatterjee, Sridar V Chittur, JoEllen Welsh, Martin P Tenniswood

**Affiliations:** 1 Department of Biomedical Sciences, University at Albany, State University of New York, Albany, NY 12222, USA; 2 Cancer Research Center, University at Albany, Rensselaer, NY 12144, USA; 3 Department of Environmental Health Sciences, University at Albany, State University of New York, Albany, NY 12222, USA

## Abstract

**Background:**

There is evidence from epidemiological and *in vitro *studies that the biological effects of testosterone (T) on cell cycle and survival are modulated by 1,25-dihydroxyvitamin D_3 _(1,25(OH)_2_D_3_) in prostate cancer. To investigate the cross talk between androgen- and vitamin D-mediated intracellular signaling pathways, the individual and combined effects of T and 1,25(OH)_2_D_3 _on global gene expression in LNCaP prostate cancer cells were assessed.

**Results:**

Stringent statistical analysis identifies a cohort of genes that lack one or both androgen response elements (AREs) or vitamin D response elements (VDREs) in their promoters, which are nevertheless differentially regulated by both steroids (either additively or synergistically). This suggests that mechanisms in addition to VDR- and AR-mediated transcription are responsible for the modulation of gene expression. Microarray analysis shows that fifteen miRNAs are also differentially regulated by 1,25(OH)_2_D_3 _and T. Among these miR-22, miR-29ab, miR-134, miR-1207-5p and miR-371-5p are up regulated, while miR-17 and miR-20a, members of the miR-17/92 cluster are down regulated. A number of genes implicated in cell cycle progression, lipid synthesis and accumulation and calcium homeostasis are among the mRNA targets of these miRNAs. Thus, in addition to their well characterized effects on transcription, mediated by either or both cognate nuclear receptors, 1,25(OH)_2_D_3 _and T regulate the steady state mRNA levels by modulating miRNA-mediated mRNA degradation, generating attenuation feedback loops that result in global changes in mRNA and protein levels. Changes in genes involved in calcium homeostasis may have specific clinical importance since the second messenger Ca^2+ ^is known to modulate various cellular processes, including cell proliferation, cell death and cell motility, which affects prostate cancer tumor progression and responsiveness to therapy.

**Conclusions:**

These data indicate that these two hormones combine to drive a differentiated phenotype, and reinforce the idea that the age dependent decline in both hormones results in the de-differentiation of prostate tumor cells, which results in increased proliferation, motility and invasion common to aggressive tumors. These studies also reinforce the potential importance of miRNAs in prostate cancer progression and therapeutic outcomes.

## Background

Prostate cancer is the most commonly diagnosed non-cutaneous cancer in American males and is the second leading cause of cancer-related deaths in males in North America [1]. Androgens, including testosterone (T) and its active metabolite 5α-dihydrotestosterone (5α-DHT), are important for the development and growth of early stage prostate tumors and exert their effects via androgen receptor (AR) [2-4]. Androgen ablation has been one of the mainstays for the treatment of early stage, organ-confined prostate cancer along with surgery and radiation therapy.

Several epidemiological studies have suggested that adequate levels of vitamin D are critical for the prevention of various solid tumors, including breast, ovarian and colon cancers [5,6]. The risk of developing and dying of these cancers appears to be inversely correlated with sun exposure, and/or vitamin D status, suggesting that vitamin D has chemopreventive properties [7]. Some studies have also suggested that vitamin D may play a role in prostate cancer prevention [8,9], but the data are less convincing than in other tumors and several recent meta-analyses have found weak or no associations between serum 25-hydroxyvitamin D_3 _(25(OH)_2_D_3_) levels and tumor incidence and progression [10-13]. In addition, the effects of 1,25(OH)_2_D_3 _on tumor growth in the TRAMP, LPB-Tag transgenic and Nkx3.1;PTEN mutant mouse models of prostate cancer have produced conflicting results [14-16]. However, a variety of *in vitro *studies demonstrate that 1,25(OH)_2_D_3 _or its non-calcemic analogs (EB1089; CB 1093; Gemini analogs) induce apoptosis in a variety of prostate cancer cell lines including LNCaP, LNCaP C4-2, ALVA-3, LAPC-4, DU-145 and PC-3 [17-20]. These effects appear to occur through a combination of G_0_/G_1 _cell cycle arrest, apoptosis, differentiation and inhibition of angiogenesis [21-25]. In contrast, other studies have shown that 1,25(OH)_2_D_3 _induces cell cycle arrest but not apoptosis [26-28]. These disparate effects of 1,25(OH)_2_D_3 _on prostate tumor biology appear to be dictated predominantly by the androgen status of the mice [16] or the level of androgen in the culture medium [17,29], suggesting that in prostate cancer, there may be significant cross talk between androgen-mediated growth and vitamin D_3_-mediated cell cycle arrest and differentiation which may influence tumor initiation and progression, and impact tumor growth and affect subsequent therapeutic intervention [17].

MicroRNAs (miRNAs) are a class of small non-coding, single-stranded RNAs that post-transcriptionally modulate the steady state levels of mRNA by targeting the 3' untranslated regions (3'UTR) of mRNAs. Recent studies have found that aberrant miRNA expression is closely associated with prostate cancer initiation and progression [30,31]. Several miRNAs that possess either oncogenic (miR-221/222, miR-21, miR-125b) [32-36] or tumor suppressor roles (miR-34 cluster, miR-146a, miR-200c) [37,38] have been identified in prostate cancer and some of these are associated with the castration resistant phenotype [34], or hormone-independent growth of prostate cancer [33]. Neither the effect of 1,25(OH)_2_D_3 _on miRNA levels in prostate cancer cell lines, nor the interaction with androgen signaling to modulate mRNA and miRNA transcription have been investigated. However, the importance of a regulatory loop involving miR-106b and p21 mRNA which is modulated by 1,25(OH)_2_D_3 _in non malignant prostate cells has recently been described [39]. The experiments described in this manuscript examine effects of testosterone and 1,25(OH)_2_D_3_, administered alone or in combination, on the mRNA and miRNA expression in LNCaP cells and demonstrate that cross talk between VDR- and AR-mediated signaling significantly influences the biology of prostate cancer cells. Using concurrent microarray analyses in LNCaP cells of both miRNA and mRNA, we have found that androgen-mediated transcription of both mRNA and miRNA is enhanced by 1,25(OH)_2_D_3_, either additively or synergistically, highlighting the extensive cross talk between the two receptors. Many of the gene targets of T and 1,25(OH)_2_D_3 _have significant clinical relevance. The data demonstrate that while androgens may play a central role in the development of prostate cancer, declining T levels common in older patients may play a significant role in tumor progression, particularly in patients who are also vitamin D deficient.

## Results

### Biological Response of LNCaP cells to T and 1,25(OH)_2_D_3_

**Figure 1:**
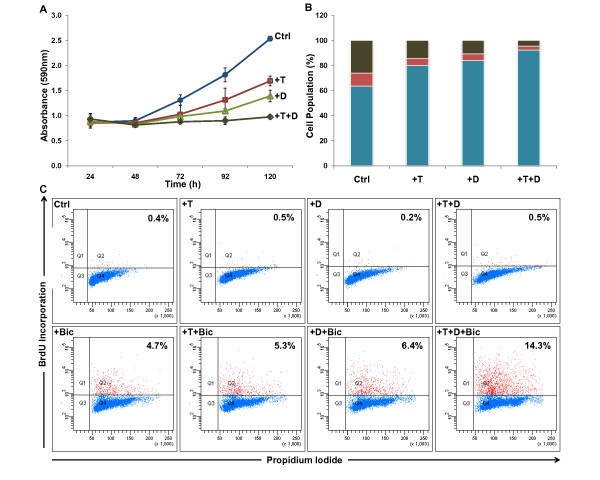
**The effect of T and 1,25(OH)_2_D_3 _alone and in combination on LNCaP cells**. LNCaP cells were plated as described in Material and Methods, and treated with 5 nM T and 100 nM 1,25(OH)_2_D_3 _alone or in combination for the indicated times (Panel A) or 48 h (panels B and C). *Panel A*: changes in cell number were measured by crystal violet; *Panel B*: cell cycle kinetics were measured by flow cytometry using propidium iodide staining (Blue:G_0_/G_1 _phase, Red: S phase; Black G_2_/M); *Panel C*: Cell death was monitored by flow cytometry of Apo-BrdU incorporation as described in Experimental Procedures [(Apoptotic cells shown upper right quadrant (Q2)]. LNCaP cells were also treated with bicalutamide in the absence and presence of T and 1,25(OH)_2_D_3 _for 48 h.

1,25(OH)_2_D_3 _has variously been reported to induce G_0_/G_1 _cell cycle arrest or apoptosis in androgen-responsive LNCaP cells and other cell lines. In our hands, 100 nM 1,25(OH)_2_D_3 _and 5 nM T alone reduce growth of LNCaP cells as measured by crystal violet staining (Figure 1A). This correlates to the induction of G_0_/G_1 _cell cycle arrest in LNCaP cells (Figure 1B) with no evidence of apoptosis (Figure 1C). The combination of T and 1,25(OH)_2_D_3 _attenuates cell growth to a greater extent than either treatment alone (Figure 1A), which correlates to the nearly synchronous arrest of the cell populations in the G_0_/G_1 _phase of the cell cycle (Figure 1B). There was no evidence of cell death in LNCaP cells after treatment with 1,25(OH)_2_D_3 _alone or in combination with T, as monitored by changes in the sub G_0 _population after staining with propidium iodide (not shown) or DNA fragmentation as measured by Apo-BrdU (Figure 1C). The lack of apoptosis in these cells is not due to defects in the apoptotic machinery, since bicalutamide induces apoptosis in LNCaP cells both in the absence and presence of T and 1,25(OH)_2_D_3 _(Figure 1C). The effects of T and 1,25(OH)_2_D_3 _on these parameters have been characterized at earlier and later time points with similar results (results not shown). These data demonstrate that AR- and VDR-mediated intracellular signaling pathways cooperate to modulate cell cycle kinetics in prostate cancer cells and attenuate their growth and proliferation without directly affecting apoptosis. They also demonstrate that the combination of T and 1,25(OH)_2_D_3 _does not block the sensitivity of the cells to bicalutamide.

### Effect of T and 1,25(OH)_2_D_3 _on gene expression in LNCaP cells

**Figure 2:**
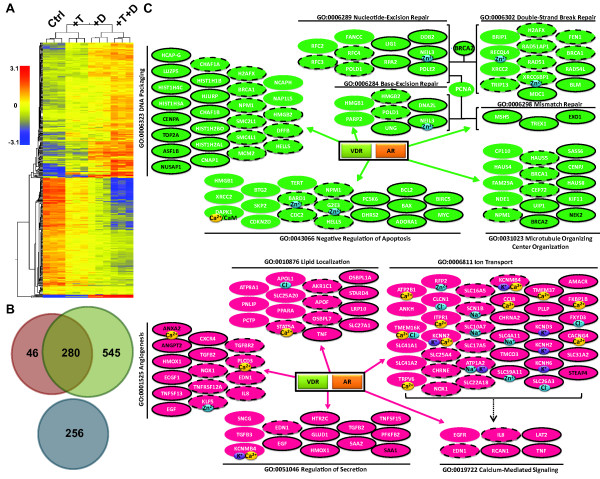
**The effect of T and 1,25(OH)_2_D_3 _on mRNA expression in LNCaP cells**. LNCaP cells were plated as described in Material and Methods, and treated with 5 nM T and 100 nM 1,25(OH)_2_D_3 _alone or in combination for 48 h. Total RNA was extracted and interrogated on Nimblegen-HG18-4plex arrays. Three independent replicates of each treatment were used for the analysis. *Panel A*: hierarchical clustering of the expression array data using the GeneSpring GX10 software. *Panel B*: Venn diagram analysis of the microarray data (Green: 1,25(OH)_2_D_3_-moduated, Orange: T-modulated, Blue: synergistically modulated genes by T and 1,25(OH)_2_D_3_. *Panel C*: Analysis of Selected Gene Ontologies Modulated by T and 1,25(OH)_2_D_3 _in LNCaP Cells. Functional annotation of each gene was assigned based on DAVID Bioinformatics Resources 2008 (NIAID). Magenta: gene up regulated by treatment; Green: down regulated by treatment; No shape outline: genes modulated by either T or 1,25(OH)_2_D_3_; dashed outline with white text: 1,25(OH)_2_D_3 _or T modulation is enhanced by the presence of T or 1,25(OH)_2_D_3 _respectively (synergy); solid outline with white text: additive effect of T and 1,25(OH)_2_D_3 _on mRNA levels; solid outline with black text: synergistic effect of T and 1,25(OH)_2_D_3 _on mRNA levels. Genes reported to be ion binding or ion channels are indicated (Ca^2+^: yellow, Cl^-^; blue, K^+^: purple, Na^+^: blue, Zn^2+^: blue).

Total RNA samples obtained from LNCaP cells treated for 48 h with 5 nM T and 100 nM 1,25(OH)_2_D_3 _alone or in combination were interrogated with Nimblegen-HG18-4plex whole genome microarrays. Gene expression profiles were clustered based on gene entities and treatment conditions. Treatment with T or 1,25(OH)_2_D_3 _alone and in combination shows distinct expression patterns that are tightly clustered by their treatment groups (Figure 2A). After filtering for the number of positive probes per gene, statistical analysis on the microarray array data with 1.5 fold cut-off generates a gene list that contains 1127 gene entities that are modulated by either T (326) or 1,25(OH)_2_D_3 _(825) or both additively (280) in LNCaP cells. Omnibus testing demonstrates that the effect of T and D on the expression of these genes is highly significant (p < 0.0001). Many of the 825 genes regulated by 1,25(OH)_2_D_3_, identified in this array have been identified as vitamin D responsive genes in other studies [40-42]. Approximately 65% of T modulated genes (202 of 326) has been previously reported to be responsive to androgens (T, 5α-dihydrotestosterone or R1881) in other *in vitro *systems (Androgen Responsive Gene Database: http://argdb.fudan.edu.cn). Thus, addition of exogenous T to the medium of LNCaP cells identifies a significant new cohort of 124 mRNAs that are androgen responsive. Furthermore, T and 1,25(OH)_2_D_3 _also synergistically modulate 256 genes that are not significantly regulated by either hormone alone, and thus form a nonintersecting dataset (Figure 2B). These data suggest AR and VDR share many common gene targets and cooperate to regulate cellular processes in LNCaP cells.

Gene Set Enrichment Analysis using Pathways Studio and Gene Ontology analysis from DAVID Bioinformatics Resources (NIAID) were used to assess the significance of the interactions between T and 1,25(OH)_2_D_3 _in LNCaP cells. As shown in Table 1, T alone significantly affects processes associated with cell division (particularly mitosis), microtubule based movement, chromosome segregation and progression through anaphase in LNCaP cells. 1,25(OH)_2_D_3 _alone also significantly affects the expression of genes associated with these cellular processes, in addition to those involved in calcium ion homeostasis and phosphoinositide-mediated signaling. Treatment with T and 1,25(OH)_2_D_3 _enhances the response of genes associated with these ontologies, and, as revealed by comparing Figure 2C and additional files 1 and 2, the combination of the two hormones also additively or synergistically modulates a significantly greater number of genes than either hormone alone. It is evident that T and 1,25(OH)_2_D_3 _individually modulate the expression of many of the genes in these ontologies while the combination of the two hormones modulates a significant number of additional genes.

**Table 1:** Gene Set Enrichment Analysis of representative gene sets identified as significantly enriched after 1,25(OH)_2_D_3 _treatment in the presence of androgen in LNCaP cells.

Biological Process	Testosterone		1,25(OH)_2_D_3_		T & D	
	
	Median Change	p-value	Median Change	p-value	Median Change	p-value
**Mitosis**	-1.54	3.51E-10	-1.98	3.63E-07	-3.63	1.27E-15

**Microtubule-based movement**	-1.62	2.54E-05	-2.11	1.17E-04	-4.19	5.78E-07

**Chromosome segregation**	-1.61	1.11E-03	-1.99	2.13E-03	-3.44	1.12E-04

**Anaphase-promoting complex-dependent proteasomal ubiquitin-dependent protein catabolic process**	-1.47	5.90E-03	n/a	n/a	-3.63	2.10E-03

**DNA repair**	n/a	n/a	-1.77	1.08E-02	-2.55	2.24E-03

**DNA recombination**	n/a	n/a	-2.02	4.30E-02	-3.42	1.41E-02

**Phosphoinositide-mediated signaling**	n/a	n/a	-1.73	4.97E-02	-2.40	1.60E-02

**Elevation of cytosolic calcium ion concentration**	n/a	n/a	2.02	1.76E-02	3.43	2.95E-02

(False Discovery Rate <5%)

### qPCR Validation of Microarray Analyses

**Figure 3:**
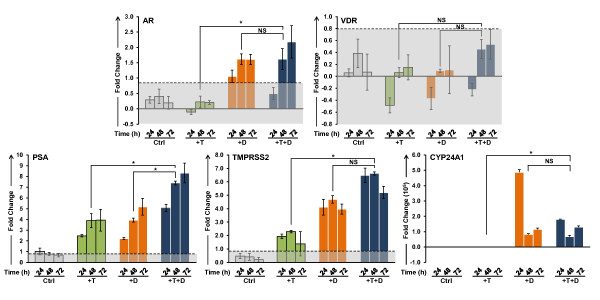
**Validation of changes in the mRNA levels of AR, VDR, PSA, TMPRSS2 and CYP24A1**. LNCaP cells were treated with 5 nM T and 100 nM 1,25(OH)_2_D_3 _alone and in combination and transcript levels were measured by Real Time-PCR over a 72 h time course. Fold changes greater than 0.8 are statistically significant; values within the shaded areas in each graph are not significantly modulated (0.8 or below after transformation). Comparisons between different treatment groups were analyzed using one-way ANOVA; differences were considered significant if p < 0.05 (*), NS: not significant. Significant changes (p < 0.05) in at least two out of three time points were required for the changes to be considered biologically relevant. Note: Scales on the ordinate axis vary from transcript to transcript.

**Figure 4:**
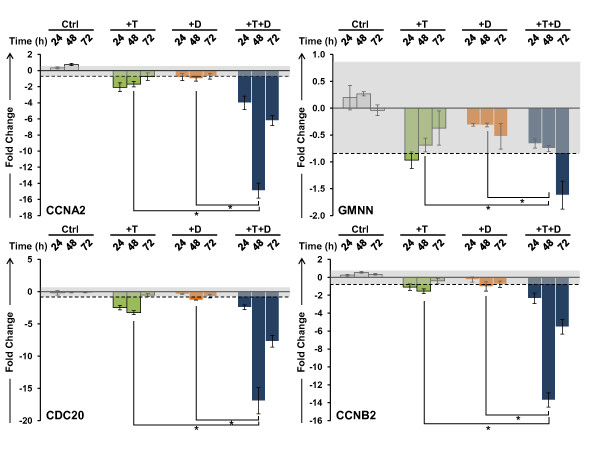
**Validation of changes in the mRNA levels of cell cycle regulators**. CCNA2, GMNN, CDC20 and CCNB2 transcript levels were measured over a 72 h time course in LNCaP cells after treatment with 5 nM T and 100 nM 1,25(OH)_2_D_3 _alone and in combination. Other details as shown in Figure 3.

**Figure 5:**
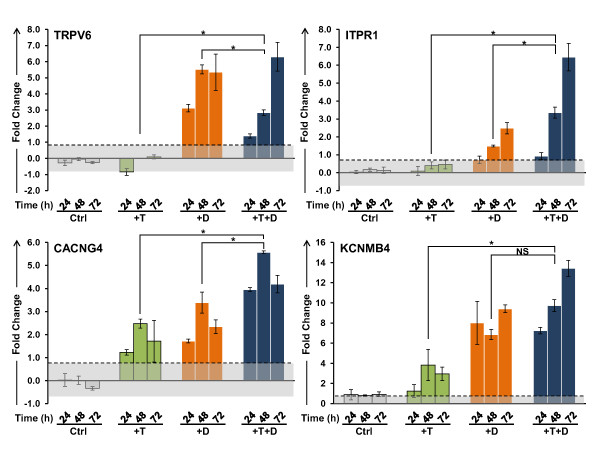
**Validation of changes in the mRNA levels of genes involved in the calcium ion homeostasis**. TRPV6, ITPR1, CACNG4 and KCNMB4 transcript levels were measured over a 72 h time course in LNCaP cells after treatment with 5 nM T and 100 nM 1,25(OH)_2_D_3 _alone and in combination. Other details as shown in Figure 3.

Validation of the microarray data of selected genes associated with the gene sets was performed in LNCaP cells after treatment with T and 1,25(OH)_2_D_3 _over a 72 h time course. The effects of these treatments on the expression of AR and VDR, as well as two well characterized androgen responsive genes, prostate specific antigen (PSA) and TMPRSS2, and CYP24A1, the classical VDR target gene are shown in Figure 3. Neither the AR nor VDR transcripts are significantly induced in LNCaP cells by T. However, 1,25(OH)_2_D_3 _alone or in combination with T increases the steady state level of AR mRNA at 48 and 72 h. This corresponds to a consistent increase in the level of the androgen receptor in the nucleus after treatment with 1,25(OH)_2_D_3 _in the absence or presence of T (additional file 3). Both hormones increase the transcript levels of PSA and TMPRSS2, and the effect of the two hormones together is additive. The classic VDR target gene CYP24A1 is strongly induced by 1,25(OH)_2_D_3_, however T alone has little or no effect on its expression. These data demonstrate that the two intracellular signaling pathways are active in LNCaP cells and that the VDR-mediated signaling significantly affects the response of both androgen responsive PSA and TMPRSS2 genes. Representative qPCR validation data, grouped by GO classification, are shown in Figure 4 (cell cycle) and Figure 5 (ion homeostasis), and the changes in the expression of genes associated with other ontologies are shown in additional file 4 (cell survival and cell death), and additional file 5 (lipid metabolism, angiogenesis, DNA repair). It is clear from these data that a number of genes are modulated by the combination of the two steroids either additively (CYP2U1, HPGD, CXCR4, CACNG4, KCNMB4, GMNN) or synergistically (CCNA2, CDC20, CCNB2, Survivin/BIRC5, GADD45G, E2F1, ITPR1, BRCA1). Genes that are known to modulate the cell cycle (CCNA2, CDC20, CCNB2) are down regulated by T and 1,25(OH)_2_D_3 _in a similar manner, reaching a nadir at 48 h after treatment (Figure 4). T and 1,25(OH)_2_D_3 _also display prolonged effects on the steady state levels of the transcripts when administered together and the transcript levels of the genes involved in prostaglandin and lipid metabolism (CYP2U1 and HPGD), and calcium homeostasis (TRPV6, ITPR1, and KCNMB4) show time-dependent increases throughout the 72 h time course (Figure 5).

It is well established that ligand-activated AR and VDR bind to cognate response elements (ARE and VDRE respectively) to modulate gene transcription and thus the coordination between T and 1,25(OH)_2_D_3 _may be the consequence of receptor mediated transcription by both AR and VDR. While *in silico *searches for ARE and VDREs within 10 kb upstream or downstream of the structural genes shown to be modulated by T or 1,25(OH)_2_D_3 _or both, demonstrate that many of the genes have canonical response elements in their promoters (additional file 6), nearly 50% of the genes identified in the expression microarray analysis appear to lack functional hormone response elements (either ARE or VDRE or both) in their promoters (additional file 6). For instance, genes that have previously been documented to be only 1,25(OH)_2_D_3 _inducible and/or contain VDRE at their promoters (KCNMB4, CXCR4) are also modulated by T (Figures 5 and additional file 4). Furthermore, genes such as CDC20 that lack both VDREs and AREs are down regulated by 1,25(OH)_2_D_3 _and T together while neither steroid alone has significant effects on gene expression (Figures 4 and additional file 5). These data demonstrate that 1,25(OH)_2_D_3 _has distinct effects on the regulation of transcript levels and that for a significant proportion the genes, the effects of 1,25(OH)_2_D_3 _require the presence of T for maximal effect. Furthermore, it appears that in addition to modulating transcription of the responsive genes, T and 1,25(OH)_2_D_3 _modulate the stability of the transcripts via modulation of miRNA expression.

### Effect of T and 1,25(OH)_2_D_3 _on miRNA expression

**Figure 6:**
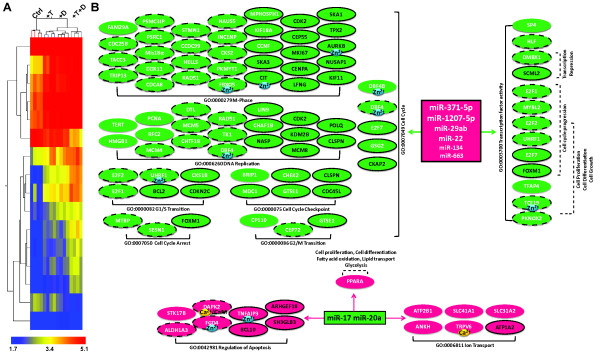
**The effect of T and 1,25(OH)_2_D_3 _on miRNA expression in LNCaP cells**. Cells were plated as described in Material and Methods, and treated with 5 nM T and 100 nM 1,25(OH)_2_D_3 _alone or in combination for 48 h. Total RNA was extracted and interrogated on Agilent Human miRNA v3 arrays. Three independent replicates were used for the analysis. *Panel A*: hierarchical clustering of the miRNA array data using the GeneSpring GX10 software. *Panel B*: Gene Ontology analysis of mRNA targets of miRNAs, predominantly focused on those that are targeted at the 3'UTR and affect the transcript stability. The inverse correlations of miRNAs targeted mRNAs and their associated Gene Ontology grouping are summarized and color coded. Magenta: gene up regulated by treatment; Green: down regulated by treatment; No shape outline: genes modulated by either T or 1,25(OH)_2_D_3_; dashed outline with white text: 1,25(OH)_2_D_3 _or T modulation is enhanced by the presence of T or 1,25(OH)_2_D_3 _respectively (synergy); solid outline with white text: additive effect of T and 1,25(OH)_2_D_3 _on mRNA levels; solid outline with black text: synergistic effect of T and 1,25(OH)_2_D_3 _on mRNA levels. Genes reported to be ion binding or ion channels are indicated (Ca^2+^: yellow, Zn^2+^: blue). Different font size of miRNAs ranks the effect of miRNAs by the number of concordant mRNA targets in LNCaP cells.

To examine the effect of T and 1,25(OH)_2_D_3 _on miRNA expression, total RNA prepared from LNCaP cells 48 h after treatment using the same experimental paradigm outlined above was analyzed on the Agilent Human miRNA microarray v3, which contains 866 human miRNAs from the Sanger database v12.0. The miRNA expression profiles in LNCaP cells after treatment cluster to the different treatment groups are shown in Figure 6A. Statistical analysis with a stringent cut-off of 2.0 fold, identifies 15 miRNAs that are significantly modulated by T and 1,25(OH)_2_D_3 _(Table 2). The majority of miRNAs identified are up regulated by T and 1,25(OH)_2_D_3 _additively, including miR-29ab and miR-371/373 clusters. In contrast, two miRNAs, including miR-17 and miR-20a, members of the miR-17/92 cluster, are down regulated by T and 1,25(OH)_2_D_3 _synergistically. Bioinformatic analysis using the available miRNA target prediction databases (TargetScan Human v5.1) identifies approximately 8500 putative mRNA targets of these miRNAs. However, most of these targets are not expressed in the prostate and are not identified as differentially regulated by T and 1,25(OH)_2_D_3 _by microarray. In total, 264 target transcripts are responsive to T and 1,25(OH)_2_D_3 _in LNCaP cells and show an inverse association with the targeting miRNA(s) (Figure 6B). This corresponds to approximately 23% of the genes modulated by T and 1,25(OH)_2_D_3 _in LNCaP cells. However this is at best a rough estimate since many of the targets of the modulated miRNAs identified by Target Scan have not yet been validated in LNCaP cells. A complete list of the miRNAs and their mRNA targets is provided in additional file 7.

**Table 2:** miRNAs differentially modulated by 1,25(OH)_2_D_3 _and T alone or in combination in LNCaP cells, identified by Agilent Human miRNA microarray v3.

miRNA	Seed	Testosterone	1,25(OH)_2_D_3_	T + 1,25(OH)_2_D_3_
**hsa-miR-17**	aaagug	-1.237	-1.255	-2.337

**hsa-miR-20a**	aaagug	-1.220	-1.287	-2.318

**hsa-miR-20b**	aaagug	-1.174	-1.391	-2.273

**hsa-miR-542-5p**	cgggga	-1.232	2.304	1.197

**hsa-miR-29b**	agcacc	1.317	1.911	2.081

**hsa-miR-1207-5p**	ggcagg	1.756	1.637	2.242

**hsa-miR-22**	agcugc	1.691	1.745	2.312

**hsa-miR-1915**	cccagg	2.005	2.044	3.007

**hsa-miR-29a**	agcacc	2.122	2.577	3.108

**hsa-miR-371-5p**	cucaaa	1.918	2.163	3.197

**has-miR-663**	ggcggg	2.293	2.561	3.685

**hsa-miR-134**	gugacu	2.995	2.923	4.597

hsa-miR-135a*	auaggg	2.609	2.773	4.818

**hsa-miR-1181**	cgucgc	3.003	3.454	5.775

hsa-miR-629*	uucucc	3.143	3.263	6.160

The seed sequence corresponds to individual miRNAs is indicated.

**Figure 7:**
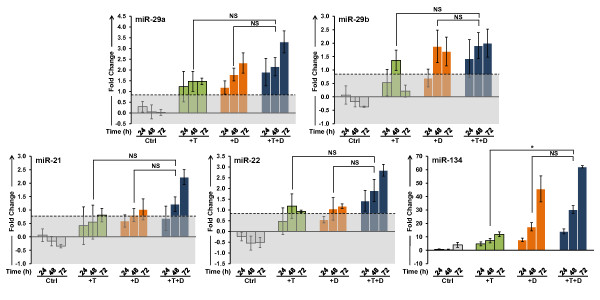
**Validation of induction of steady state levels of miR-29a, miR-29b, miR-21, miR-22, and miR-134**. LNCaP cells were treated with 5 nM T and 100 nM 1,25(OH)_2_D_3 _alone and in combination and transcript levels were measured by TaqMan^® ^qPCR over a 72 h time course. Other details as shown in Figure 3.

**Figure 8:**
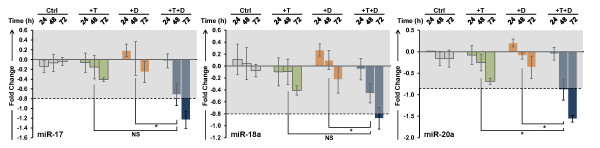
**Validation of the down regulation of steady state levels of members of the miR-17/92 cluster**. LNCaP cells were treated with 5 nM T and 100 nM 1,25(OH)_2_D_3 _alone and in combination and transcript levels were measured by TaqMan^® ^qPCR over a 72 h time course. Other details as shown in Figure 3.

The effects of T and 1,25(OH)_2_D_3 _on the steady state levels of selected miRNAs were further assessed in LNCaP cells by TaqMan^® ^qPCR to validate microarray data. T and 1,25(OH)_2_D_3 _alone showed time-dependent induction of miR-29a, miR-29b, miR-21, miR-22 and miR-134 expression in LNCaP cells while the combination of the two have a more rapid additive effects on these miRNAs (Figure 7). In contrast, neither steroid alone down regulates miR-17 and miR-20a of the miR-17/92 cluster, however the combination of T and 1,25(OH)_2_D_3 _significantly down regulates miR-17 and miR-20a, demonstrating the synergistic ability of T and 1,25(OH)_2_D_3 _to modulate miRNA levels (Figure 8). We have also assessed the changes of miR-18a, another member of the miR-17/92 cluster. Changes in miR-18a transcript levels showed a similar pattern as that of miR-17 and 20a, suggesting that T and 1,25(OH)_2_D_3 _together modulate all members of the miR-17/92 cluster rather than selectively down regulating individual members of the cluster.

In addition to the miRNAs identified from the microarray analysis, we have also assessed the effect of T and 1,25(OH)_2_D_3 _on miRNAs that have been reported to be deregulated in prostate cancer. The oncogenic miR-221, which has been reported to contribute to androgen-resistance phenotype is slightly down regulated by T and 1,25(OH)_2_D_3 _at 48 h, though the fold change is not statistically significant (additional file 8). Changes in the AR-inducible [35] and VDR-inducible [43] miR-125b are also not significant in LNCaP cells in this experimental paradigm (additional file 8). However, as already shown, the steady state levels of miR-21 are up regulated by T and 1,25(OH)_2_D_3 _in LNCaP cells with the highest fold-induction at 72 h in the presence of both hormones (Figure 7). This implies that the expression of miR-21 targets, such as PDCD4, may also be modulated by AR and VDR in LNCaP cells.

## Discussion

The data presented here demonstrate that both T and 1,25(OH)_2_D_3 _modulate the mRNA and miRNA profiles in LNCaP cells, and that the combination of the two hormones modulates a significantly larger cohort of transcripts than either hormone alone. In most cases, the effects of the two hormones are additive or synergistic. Since the predicted changes in the cohort of proteins present in the cells would be expected to influence the response to therapeutic agents, this raises questions regarding the interpretation of previous *in vitro *data describing the responsiveness of prostate cancer cell lines grown in the absence of these hormones.

The time frame we have chosen for the initial microarray experiments (48 h) and the times chosen for qPCR validation (24, 48 and 72 h) were selected based on previous experiments examining the induction of apoptosis in LNCaP cells by diverse agents including bicalutamide and Iejimalide [44]. These time points will not discriminate between primary effects of T and 1,25(OH)_2_D_3 _on transcription and secondary effects which may be due to either the modulation of miRNA, and subsequent degradation of target mRNAs or cascading regulation of transcription of other genes. However, this time frame is appropriate to examine the effects of induced miRNAs on transcript stability.

Bioinformatic analysis demonstrates that T and 1,25(OH)_2_D_3 _alter the expression of genes associated with several relevant gene ontologies that clearly have the potential to significantly influence the biology of the tumor cells, their interaction with the tumor microenvironment and their response to therapeutic intervention. Perhaps the most important of these changes relate to the regulation of calcium ion homeostasis and cell cycle progression. One of the primary physiological roles of 1,25(OH)_2_D_3 _is to regulate calcium and phosphorus metabolism in bone. However, 1,25(OH)_2_D_3 _also plays an important role in cell cycle regulation in many cancers, including breast, ovarian and colon cancer, through its interaction with the VDR [6,45-48]. In LNCaP cells, 1,25(OH)_2_D_3 _induces the expression of both voltage-gated (CACNG4) and non-voltage-gated (TRPV6) Ca^2+ ^channels located on the plasma membrane. Though TRPV6 is primarily modulated by VDR, the full induction of ITPR1, annexin AII (ANXA2) and S100A10, major components of the TRPV6 auxiliary protein complex, requires the presence of both hormones, as does the induction of the highly Ca^2+ ^sensitive PLC-δ3 (PLCD3). There is also a concurrent increase in ITPR1 in response to T and 1,25(OH)_2_D_3 _which has been shown to lead to increased signaling through the PI3K/AKT pathway [49]. This suggests that in response to T and 1,25(OH)_2_D_3_, the production of IP3 will increase, triggering the release of Ca^2+ ^from endoplasmic reticulum (ER) stores via ITPR1, leading to the activation of TRPV6 through S100A10, resulting in an influx of Ca^2+ ^and augmenting intracellular Ca^2+ ^levels. In addition, diacylglycerol, produced by PLC mediated cleavage of PIP2, may activate PKC, to further increase the activity of TRPV6 via phosphorylation [50]. Together, these data suggest that the combination of T and 1,25(OH)_2_D_3 _elevates the intracellular Ca^2+ ^level to establish a new homeostatic set point without inducing cell death. Since the induction of the execution phase of apoptosis in prostate cancer cells requires the elevation of intracellular free Ca^2+ ^into the micromolar range [51], resetting the intracellular Ca^2+ ^concentration closer to this threshold may render tumor cells more sensitive to apoptosis-inducing agents including doxorubicin, bicalutamide and radiation. It is well known that uncontrolled release of Ca^2+ ^stores from the ER, for example in response to thapsigargin, initiates apoptosis via the activation of Ca^2+^-dependent caspases accompanied by release of cytochrome c through the mitochondrial permeability transition pore [52]. It is therefore likely that a fine-tuned Ca^2+ ^balance within the cellular compartments in prostate cancer cells occurs after treatment with 1,25(OH)_2_D_3 _and thus prevents Ca^2+ ^overload in the mitochondria and maintains other Ca^2+^-dependent signaling, which may include the modulation of miRNA expression [53]. Importantly, both T and 1,25(OH)_2_D_3 _appear to be required to establish the elevated homeostatic calcium levels. In this regard, the capacitative Ca^2+ ^entry that has been shown to block the development of the apoptosis resistance phenotype such as that seen in Bcl-2 over-expressing LNCaP cells [54,55] may be equivalent to the elevated homeostatic Ca^2+ ^level induced by T and 1,25(OH)_2_D_3_, suggesting that adequate levels of the two hormones should also prevent the development of the apoptosis-resistant, or castration resistant, phenotype in prostate cancer.

Many of the changes in the steady state mRNA levels can be attributed to changes in transcriptional activity due to the presence of functional AREs and/or VDREs in the promoters of target genes. However, *in silico *searches suggests that the promoters of nearly 40% of the affected genes do not contain either response element. Both T and 1,25(OH)_2_D_3 _have been shown to induce rapid Ca^2+ ^influx via store-operated Ca^2+ ^(SOC) channels whose activities are mediated by membrane receptors (mVDR & mAR) and are related to the non-genomic action of AR and VDR [56-58]. In addition, the ligand-activated VDR can modulate transcription of some target genes through Sp-1 sites [59-61]. Therefore, it is likely that additional mechanisms are responsible for the extensive modulation seen in response to the two hormones.

As demonstrated here, T and 1,25(OH)_2_D_3 _cooperatively modulate a circumscribed group of miRNAs, which mediate mRNA degradation depending on the complementarity of the miRNA seed sequence to sequences within the 3'UTR of the transcript. While alterations in individual miRNAs are not as profound as those seen in the mRNA profiles, many of the miRNAs share identical seed sequences, which results in a cumulative effect on the target transcripts. In LNCaP cells, most of the miRNAs identified in this study are up regulated in response to T and 1,25(OH)_2_D_3_, including miR-21, miR-22, miR-29ab, miR-134 and miR-371-5p and miR-1207-5p. Both miR-21 and miR-29b have previously been shown to be induced by R1881 in LNCaP and LAPC-4 prostate cancer cells [62]. Induction of these miRNAs results in substantial down regulation of several large cohorts of genes classified by GO. Thus, in addition to down regulating transcription through their cognate hormone response elements, T and 1,25(OH)_2_D_3 _can profoundly affect the stability of the transcripts encoding proteins that function in cell cycle control, cytoskeleton organization, and DNA damage repair among others. In this context, MYCBP, a positive regulator of c-Myc activity and a validated miR-22 target should be repressed by T and 1,25(OH)_2_D_3_, thus inhibiting c-Myc mediated transcription of E-box containing genes [63]. In breast cancer cells, this correlates with suppressed cell proliferation and anchorage-independent growth suggesting that increased expression of miR-22 at 48 h in prostate tumor cells by T and 1,25(OH)_2_D_3 _may be partially responsible for cell cycle arrest and the prevention of tumor progression. In LNCaP cells miR-134 is very significantly up regulated by 1,25(OH)_2_D_3 _in the absence and presence of T. The role of miR-134 in prostate cancer has not been previously described, however the steady state level of miR-134 is modulated by members of the p53/p73/p63 family as part of a miRNA-tumor suppressor network [64]. Thus, increased expression of miR-134 may further contribute to the activity of T and 1,25(OH)_2_D_3 _to suppress tumor growth.

While miR-21 was not identified in the microarray as a T and/or 1,25(OH)_2_D_3 _regulated miRNA, it was identified in the more informative qPCR analysis as a target of T and 1,25(OH)_2_D_3_, and the increases in the steady state level of miR-21 are significant after 48 h of treatment. Previous studies in breast and pancreatic cancer cell lines have suggested that over expression of miR-21 may lead to increased cell proliferation and decreased apoptosis through the targeted degradation of tumor suppressor protein PDCD4 [62,65]. In prostate cancer cells, these effects would be anticipated to counteract the anti-proliferative effects of 1,25(OH)_2_D_3_, suggesting that not all interactions between AR- and VDR-mediated signaling are beneficial. Interestingly, neither of the other miRNAs that have been implicated in prostate tumor progression (miR-221 or miR-125b) is modulated by T nor 1,25(OH)_2_D_3 _in this *in vitro *model system. In addition to up regulating miRNAs that encode cell cycle regulatory proteins and calcium ion homeostasis, T and 1,25(OH)_2_D_3 _down regulate the expression of the oncomiR cluster, miR-17/92. These data suggest that miRNAs may play important roles in T- and 1,25(OH)_2_D_3_-induced cell cycle arrest in prostate cancer cells. The concurrent analysis of mRNA and miRNA expression has demonstrated that many of the combined effects of T and 1,25(OH)_2_D_3 _are modulated by a small cohort of 15 miRNAs that are additively or synergistically regulated by the two hormones. Since the majority of men diagnosed with prostate cancer are likely to be vitamin D insufficient [66], these data may have a profound impact on our understanding of the molecular mechanisms underlying the chemopreventive and chemotherapeutic effects of vitamin D_3_. Based on the data presented here, vitamin D deficiency is likely to render tumor cells more aggressive and less sensitive to chemotherapy. Given the trend toward "active surveillance" for men diagnosed with early stage prostate cancer, understanding the cross talk between the androgen- and vitamin D-mediated cellular effects may have a significant impact on the care of men in the period between diagnosis and the initiation of treatment. If these two signaling pathways interact in tumor tissue as demonstrated here, individual variations in dietary vitamin D and/or sun exposure, as well as differences in circulating T levels, may greatly influence the rate of prostate tumor growth and the sensitivity of prostate cancer to hormone and other chemotherapies. In this context, the age related decline in serum testosterone may also contribute to the progression of prostate cancer. Thus, maintaining serum testosterone and combined with supplementation of vitamin D may substantially slow disease progression for men diagnosed with very early stage cancer, extending the time between diagnosis and treatment.

## Methods

### Cell Culture

LNCaP human prostate cancer cells, obtained from American Type Culture Collection (Rockville, MD), were grown in RPMI-1640 medium (Invitrogen, Carlsbad, CA) supplemented with 10% FBS (Sigma-Aldrich, St Louis, MO), 100 U/mL penicillin and 100 μg/mL streptomycin. Cells were maintained at 37°C in a humidified atmosphere of 95% air/5% CO_2_. For all the experiments performed in this study, with the exception of crystal violet assays, LNCaP cells were plated at a density of 1 × 10^6 ^cells per 150 cm^2 ^dish for 48 h prior to treatment with 5 nM T (Sigma-Aldrich) and 100 nM 1,25(OH)_2_D_3 _(Sigma-Aldrich) alone or in combination. The steroids were dissolved in ethanol, and control cells were treated with the same volume of vehicle.

### Crystal Violet Assay

LNCaP cells were seeded at 20,000 cells/well in 24 well plates for 24 h prior to treatment with 5 nM T and 100 nM 1,25(OH)_2_D_3 _alone or in combination. Cells were fixed with 2% glutaraldehyde in PBS for 20 min at room temperature followed by staining with 0.1% crystal violet (Sigma-Aldrich) for 30 min. The crystal violet stain was solubilized in 0.2% Triton X-100 in ddH_2_O for 30 min and the absorbance was read with Victor^3^V 1420 Multilabel Counter (PerkinElmer Inc, Waltham MA) at 590 nm. Three independent biological replicates were analyzed in triplicate.

### Flow Cytometry

LNCaP cells were treated with 5 nM T and 100 nM 1,25(OH)_2_D_3 _as described above. Cells treated with either vehicle (EtOH) or 100 μM bicalutamide serve as the negative and positive control, respectively. For cell cycle analysis, the cells were treated for 24, 48 and 72 h and harvested by trypsinization, followed by 90% ethanol permeabilization overnight at -20°C. Permeabilized cells were stained with 5 μg/mL propidium iodide (Sigma-Aldrich) in the presence of 0.015 U/mL RNase (Roche Applied Science, Indianapolis, IN) in PBS for 20 min at room temperature. Samples were analyzed within 3 h of labeling on BD™ LSR II Flow Cytometer (BD Biosciences, San Jose, CA). A minimum of 10,000 events were analyzed for each experimental condition. Three independent biological replicates for each treatment group were analyzed.

Apoptosis was analyzed using Apo-BrdU staining. Cells were harvested by trypsinization and fixed with 4% formaldehyde in PBS for 30 min on ice, followed by 70% ethanol permeabilization overnight at -20°C. Samples were enzymatically labeled with bromodeoxyuridine triphosphate in TdT reaction buffer (Br-dUTP, 2.5 mM cobalt chloride, and terminal transferase 24,000 U) for 1 h at 37°C to label the 3'-OH ends of fragmented DNA (Roche). DNA strand breaks were counterlabeled with FITC-conjugated anti-BrdU monoclonal antibody according to the manufacturer's directions (Phoenix Flow Systems, San Diego, CA). Cells were counterstained with 5 μg/mL propidium iodide for 30 min at room temperature. Samples were analyzed on BD™ LSR II Flow Cytometer (BD Biosciences) within 3 h of labeling and a minimum of 10,000 events were analyzed for each experimental condition. Three independent biological replicates for each treatment group were analyzed.

### mRNA Microarray Analysis

Total RNA isolated from LNCaP cells was processed using standard protocols for Nimblegen arrays. Briefly 10 μg of total RNA was reverse transcribed to cDNA using oligo-dT primers and Superscript II (Invitrogen), converted to double stranded cDNA and Klenow labeled with Cy3-labeled random 9-mers before hybridization to Nimblegen-Human-HG18 4 × 72 microarrays at 42°C for 16 h using a Nimblegen Hybridization system. The arrays were washed and scanned on a Genepix 4000B scanner following which the data was extracted using NimbleScan software. Further data analysis was performed using GeneSpring GX10. The raw dataset is available as a curated dataset at GEO (SuperSeries GSE23815, SubSeries GSE17461). Three independent biological replicates for each treatment group were analyzed.

### miRNA Microarray Analysis

Total RNA was processed and hybridized to Agilent Human miRNA microarrays using standard protocols. 100 ng of total RNA was dephosphorylated with calf intestinal phosphatase and end-labeled with Cy3-pCp by T4 RNA ligase prior to an overnight hybridization at 55°C onto Agilent Human miRNA v3 (Sanger release 12.0) microarrays. The arrays were washed and scanned on a high resolution GC2565CA Agilent Scanner using the manufacturer's recommended settings. The raw data was extracted using Agilent Feature Extraction software v10.1.1 and imported into GeneSpring GX10 for further analysis. The raw data is available as a curated dataset at GEO (SuperSeries GSE23815, SubSeries GSE23814).

### qPCR Validation of Microarray Data

Changes in mRNA and miRNA identified by microarray were validated in independent biological replicates. LNCaP cells were plated and treated as previously described for 24, 48 and 72 h. Cells were harvested by trypsinization and total RNA (both mRNA and miRNA) was extracted using miRNeasy mini kit (Qiagen, Valencia, CA). Reverse transcription PCR reactions were performed with 1.5 μg total RNA using Taqman^® ^Reverse Transcription Reagents (Applied Biosystems, Carlsbad, CA) to synthesize cDNA for mRNA expression analysis. The reaction mixture was incubated for 10 min at 25°C, 1 h at 37°C and 5 min at 95°C and kept at -20°C until further analysis. qPCR probes for each gene were designed using Primer-Blast (National Center for Biotechnology Information) with default settings and synthesized by Integrated DNA Technologies (Coralville, IA). The list of primers for each gene is available in additional file 9. SYBR Green reactions with SYBR^® ^Green PCR Master Mix (Applied Biosystems) were analyzed using the ABI 7900HT Fast Real-Time PCR System (Applied Biosystems): 50°C for 2 min, 95°C for 10 min, 95°C for 15 sec and 60°C for 1 min, repeated for 40 cycles. Relative expression levels of each gene in real time were analyzed using the 2^-ΔΔC^_T _method [67] and presented as ratio relative to the expression of the housekeeping gene GAPDH. For miRNA expression analysis, 10 ng total RNA were used to make cDNA with TaqMan^® ^MicroRNA Reverse Transcription Reagents (Applied Biosystems). The reaction mixture was incubated for 30 min at 16°C, 30 min at 42°C and 5 min at 85°C and kept at -20°C until further analysis. TaqMan^® ^MicroRNA Assays were used to evaluate miRNA expression according to manufacturer's protocol. TaqMan reactions were analyzed using the ABI 7900HT Fast Real-Time PCR System: 95°C for 10 min, 95°C for 15 sec and 60°C for 60 sec repeated for 40 cycles. Relative expression levels of each miRNA in real time were analyzed using the 2^-ΔΔC^T method with U6 snRNA as the reference control. Each sample was replicated twice from three independent sets of RNA preparations. Results are tabulated as mean ± SD and presented as fold change after transformation to show divergence from no effect (zero fold change).

### Statistical Analysis of Microarray Data

For mRNA microarray analysis in GeneSpring GX10, the gene list was filtered to exclude entities which showed low signal values across all samples (i.e bottom 20^th ^percentile). Statistically significant genes from each expression profile were selected using one-way ANOVA (p ≤ 0.05). The multiple testing correction Benjamini and Hochberg false discovery rate (FDR) (p-value <0.05) was integrated within each test. A fold change cut-off at 1.5 fold was implemented to generate the final list of differentially expressed genes. Genes passing the statistical tests were further assigned into their gene ontology (GO) grouping using DAVID Bioinformatics Resources v6.7 (NIAID) with the default setting, essentially as previously described [68]. Significantly regulated and enriched GO groups were selected for qPCR analysis.

For miRNA microarray analysis, the raw data was imported into GeneSpring GX10, log2 transformed, normalized to the 75^th ^percentile following which the entity list was filtered to exclude probes that showed low signal values across all treatment groups (i.e. bottom 20^th ^percentile). This list was further filtered to only include entities that were marked "present;' or "marginal" in all 3 replicates for at least one of the 4 treatment groups. Entities with fold changes greater than 2.0 were considered significant when p < 0.05 using one-way ANOVA with Benjamini-Hochberg FDR post-test. Targets of miRNAs that passed the statistical test were identified using TargetScan Human v5.1 from Whitehead Institute for Biomedical Research. Gene targets whose mRNA expression levels were inversely proportional to the corresponding miRNA expression levels were considered concordant and were further analyzed for their enrichment in Gene Ontology grouping.

## Competing interests

The authors declare that they have no competing interests.

## Authors' contributions

WWW was responsible for execution of the experiments described in this manuscript and the analysis of the data. She also wrote the first draft of the manuscript. NC assisted with the analysis of the microarray data and SVC performed the microarray experiments and the initial bioinformatic analyses. JW was responsible for experimental design and editing the manuscript. MT was responsible for experimental design and editing the manuscript. All authors read and approved the final manuscript.

## Supplementary Material

Additional file 1 **Analysis of Selected Gene Ontologies Modulated by T in LNCaP Cells**. Functional annotation of each gene was assigned based on DAVID Bioinformatics Resources 2008 (NIAID). Magenta: gene up regulated by treatment; Green: down regulated by treatment; No shape outline: genes modulated by either T or 1,25(OH)_2_D_3_; dashed outline with white text: 1,25(OH)_2_D_3 _or T modulation is enhanced by the presence of T or 1,25(OH)_2_D_3 _respectively (synergy); solid outline with white text: additive effect of T and 1,25(OH)_2_D_3 _on mRNA levels; solid outline with black text: synergistic effect of T and 1,25(OH)_2_D_3 _on mRNA levels. Genes reported to be ion binding or ion channels are indicated (Ca^2+^: yellow, Zn^2+^: blue) Click here for file

Additional file 2 **Analysis of Selected Gene Ontologies Modulated by 1,25(OH)_2_D_3 _in LNCaP Cells**. Functional annotation of each gene was assigned based on DAVID Bioinformatics Resources 2008 (NIAID). Magenta: gene up regulated by treatment; Green: down regulated by treatment; No shape outline: genes modulated by either T or 1,25(OH)_2_D_3_; dashed outline with white text: 1,25(OH)_2_D_3 _or T modulation is enhanced by the presence of T or 1,25(OH)_2_D_3 _respectively (synergy); solid outline with white text: additive effect of T and 1,25(OH)_2_D_3 _on mRNA levels; solid outline with black text: synergistic effect of T and 1,25(OH)_2_D_3 _on mRNA levels. Genes reported to be ion binding or ion channels are indicated (Ca^2+^: yellow, Zn^2+^: blue) Click here for file

Additional file 3 **Immunoblotting analysis of 5 nM T and 100 nM 1,25(OH)_2_D_3 _on nuclear AR and VDR expression**. LNCaP cells were treated with 5 nM T and 100 nM 1,25(OH)_2_D_3 _alone and in combination for 48 h. Nuclear proteins were extracted and ran on 10% SDS-PAGE and transferred to PVDF membrane. Antibodies against AR (Millipore) and VDR (Santa Cruz) were used to detect the protein levels of AR and VDR in the nucleus. Lamin A/C was used as the loading control. Click here for file

Additional file 4 **Validation on changes in the mRNA levels of genes involved in cell death**. GADD45G, STK17B, E2F1 and Survivin/BIRC5 transcript levels were measured over a 72 h time course in LNCaP cells after treatment with 5 nM T and 100 nM 1,25(OH)_2_D_3 _alone and in combination. Other details as shown in Figure 3. Click here for file

Additional file 5 **Validation on changes in the mRNA levels of selected genes involved in lipid metabolism, angiogenesis, calcium induced signaling and DNA repair**. CYP2U1, HPGD, CXCR4, ANXA2, EGFR and BRCA1 transcripts were measured over a 72 h time course in LNCaP cells after treatment with 5 nM T and 100 nM 1,25(OH)_2_D_3_. Fold changes greater than 0.8 are statistically significant; values within the shaded areas in each graph are not significantly modulated (0.8 or below after transformation). Comparisons between different treatment groups were analyzed using one-way ANOVA; differences were considered significant if p < 0.05 (*), NS: not significant. Significant changes (p < 0.05) in at least two out of three time points were required for the changes to be considered biologically relevant. Note: Scales on the ordinate axis vary from transcript to transcript. Click here for file

Additional file 6 **Differentially regulated mRNAs by T and 1,25(OH)_2_D_3 _in LNCaP cells after 48 h of treatment**. Down regulated mRNAs are shaded in green and up regulated mRNAs are shaded in red. The presence (1) or absence (0) of hormone response elements are indicated based on the *in silico *searches for androgen response elements (AREs) and/or vitamin D response elements (VDREs) in the promoters of T and 1,25(OH)_2_D_3 _responsive genes identified from microarray analysis. Click here for file

Additional file 7 **miRNA targeted that are differentially regulated by T and 1,25(OH)_2_D_3 _in LNCaP cells after 48 h of treatment**. Down regulated mRNAs/miRNAs are shaded in green and up regulated mRNAs/miRNAs are shaded in red. Click here for file

Additional file 8 **Effects of T and 1,25(OH)_2_D_3 _on the expression of miR-125b and miR-221 in LNCaP cells**. LNCaP cells were treated with 5 nM T and 100 nM 1,25(OH)_2_D_3 _alone and in combination and transcript levels were measured by TaqMan^® ^qPCR over a 72 h time course. Other details as shown in Figure S4. Click here for file

Additional file 9 **Sequence of Primers used for qPCR**. Click here for file
