# Supplementary figures and images for: Effects of 1α,25 dihydroxyvitamin D3 and testosterone on miRNA and mRNA expression in LNCaP cells
Source: Mol Cancer. 2011 May 18;10:58. doi: 10.1186/1476-4598-10-58 (PMC3112430; doi:10.1186/1476-4598-10-58)

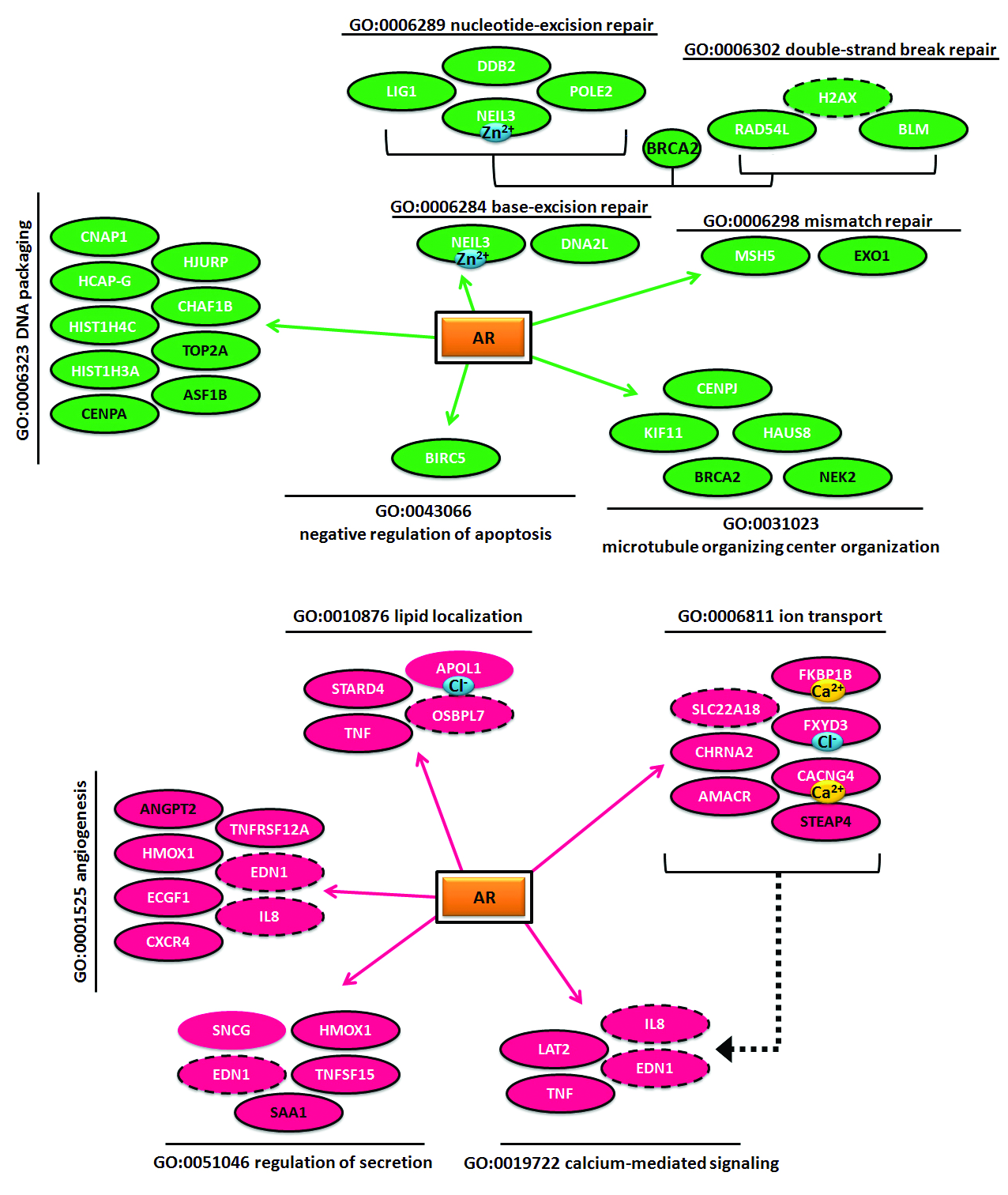

Supplement: Additional file 1 — Analysis of Selected Gene Ontologies Modulated by T in LNCaP Cells. Functional annotation of each gene was assigned based on DAVID Bioinformatics Resources 2008 (NIAID). Magenta: gene up regulated by treatment; Green: down regulated by treatment; No shape outline: genes modulated by either T or 1,25(OH)2D3; dashed outline with white text: 1,25(OH)2D3 or T modulation is enhanced by the presence of T or 1,25(OH)2D3 respectively (synergy); solid outline with white text: additive effect of T and 1,25(OH)2D3 on mRNA levels; solid outline with black text: synergistic effect of T and 1,25(OH)2D3 on mRNA levels. Genes reported to be ion binding or ion channels are indicated (Ca2+: yellow, Zn2+: blue) [file 1476-4598-10-58-S1.TIFF]

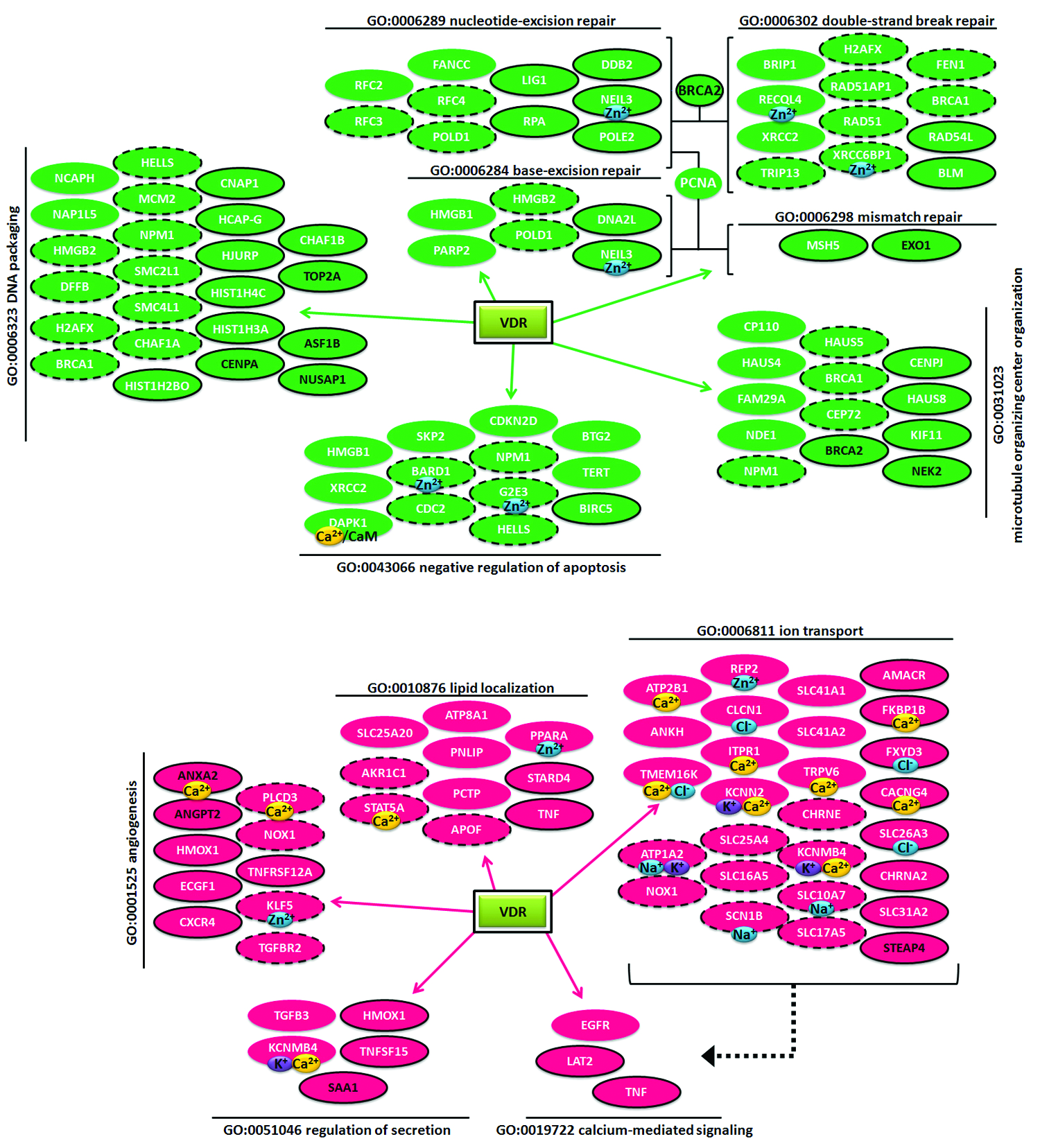

Supplement: Additional file 2 — Analysis of Selected Gene Ontologies Modulated by 1,25(OH)2D3 in LNCaP Cells. Functional annotation of each gene was assigned based on DAVID Bioinformatics Resources 2008 (NIAID). Magenta: gene up regulated by treatment; Green: down regulated by treatment; No shape outline: genes modulated by either T or 1,25(OH)2D3; dashed outline with white text: 1,25(OH)2D3 or T modulation is enhanced by the presence of T or 1,25(OH)2D3 respectively (synergy); solid outline with white text: additive effect of T and 1,25(OH)2D3 on mRNA levels; solid outline with black text: synergistic effect of T and 1,25(OH)2D3 on mRNA levels. Genes reported to be ion binding or ion channels are indicated (Ca2+: yellow, Zn2+: blue) [file 1476-4598-10-58-S2.TIFF]

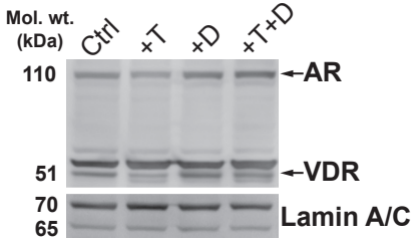

Supplement: Additional file 3 — Immunoblotting analysis of 5 nM T and 100 nM 1,25(OH)2D3 on nuclear AR and VDR expression. LNCaP cells were treated with 5 nM T and 100 nM 1,25(OH)2D3 alone and in combination for 48 h. Nuclear proteins were extracted and ran on 10% SDS-PAGE and transferred to PVDF membrane. Antibodies against AR (Millipore) and VDR (Santa Cruz) were used to detect the protein levels of AR and VDR in the nucleus. Lamin A/C was used as the loading control. [file 1476-4598-10-58-S3.PDF]

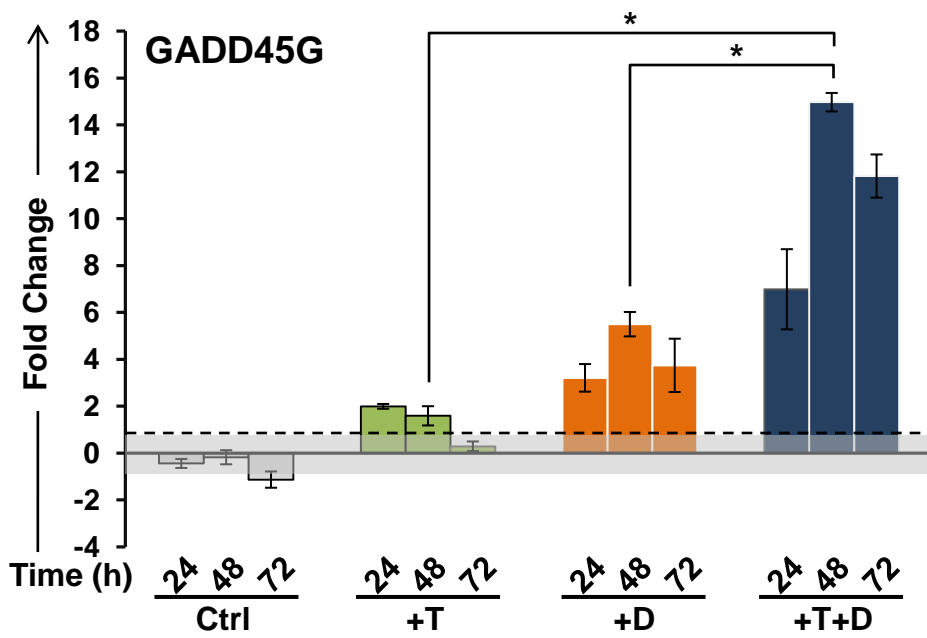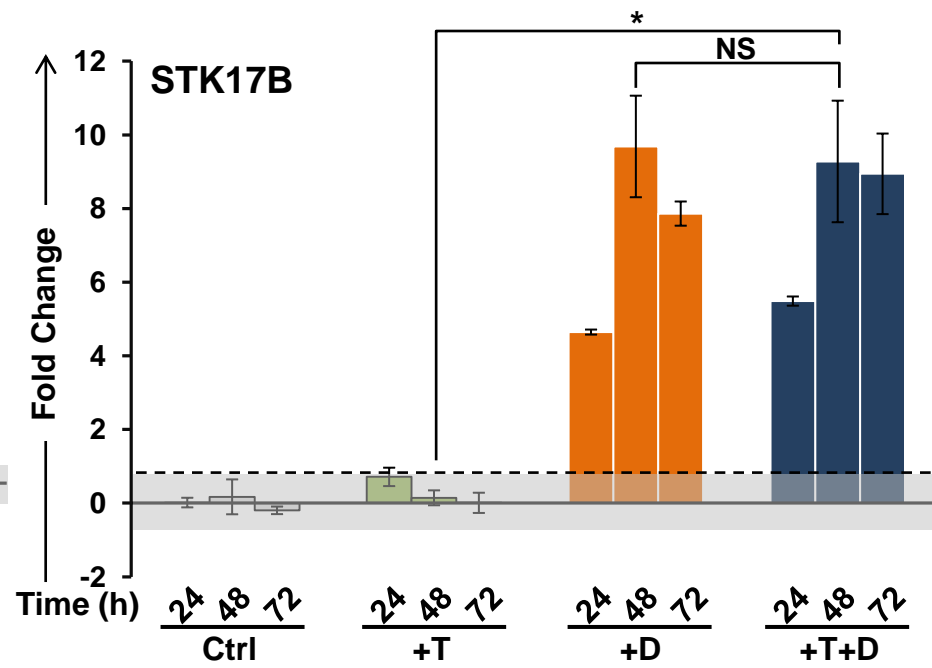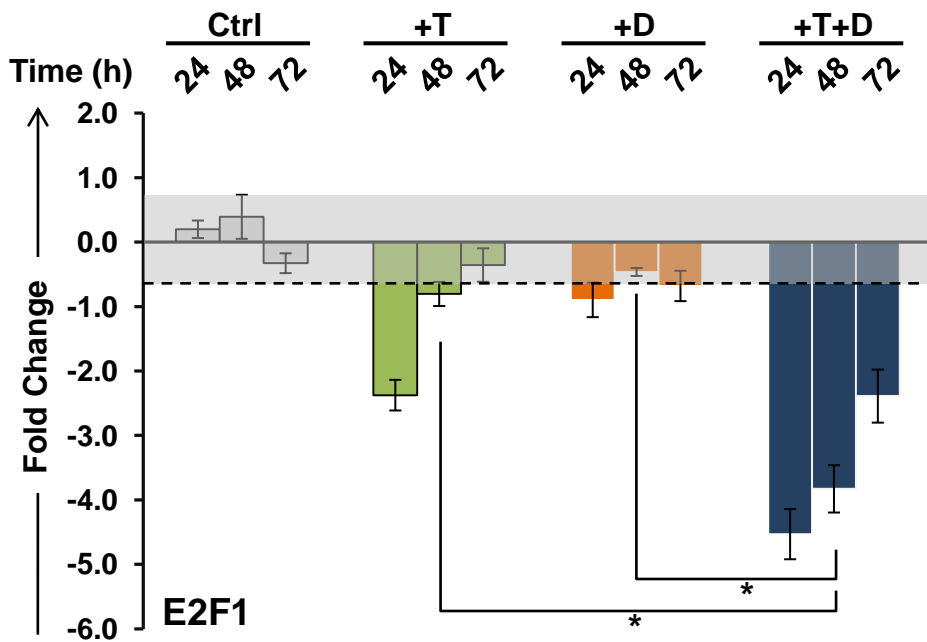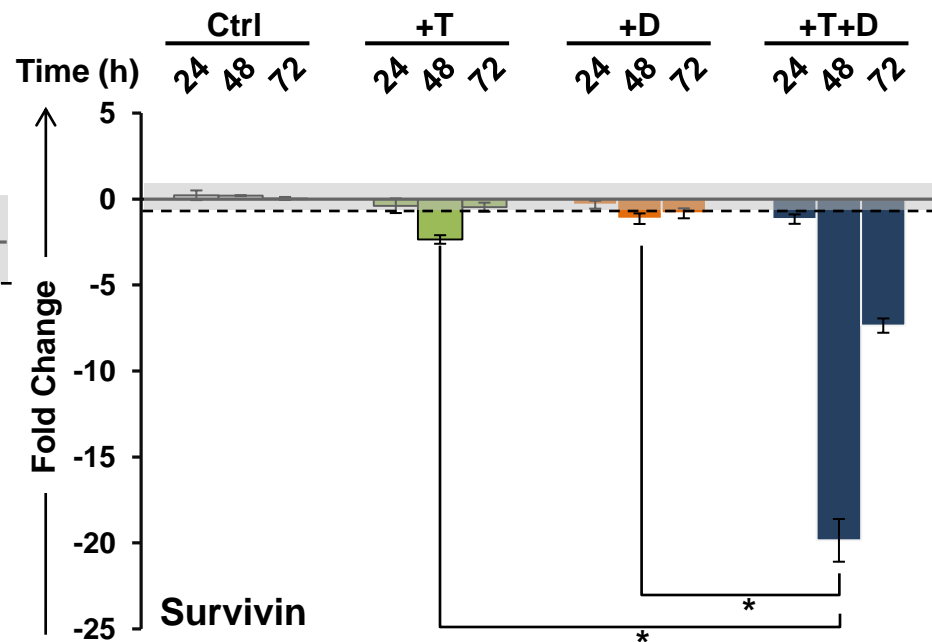

Supplement: Additional file 4 — Validation on changes in the mRNA levels of genes involved in cell death. GADD45G, STK17B, E2F1 and Survivin/BIRC5 transcript levels were measured over a 72 h time course in LNCaP cells after treatment with 5 nM T and 100 nM 1,25(OH)2D3 alone and in combination. Other details as shown in Figure 3. [file 1476-4598-10-58-S4.PDF]

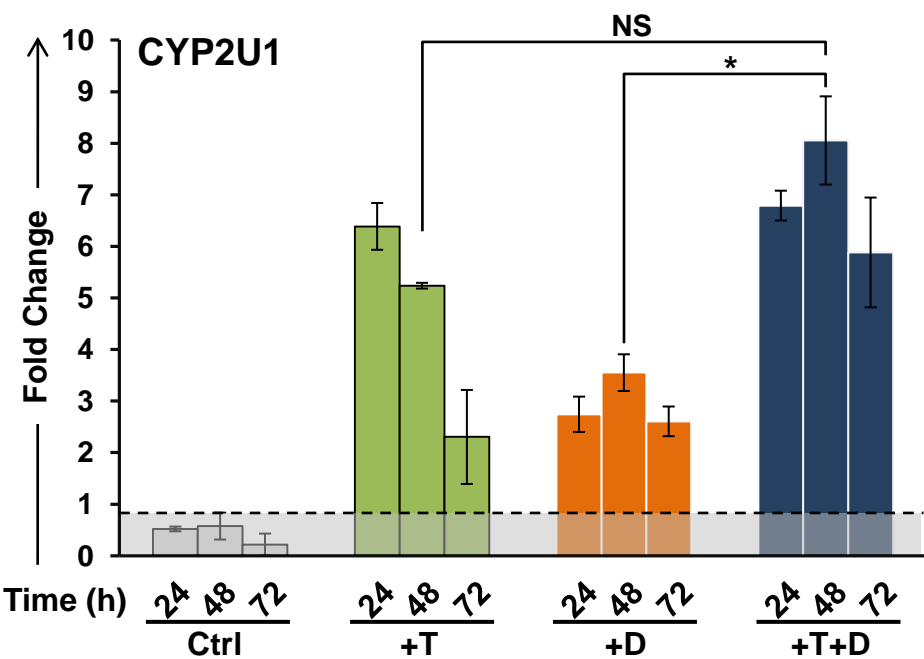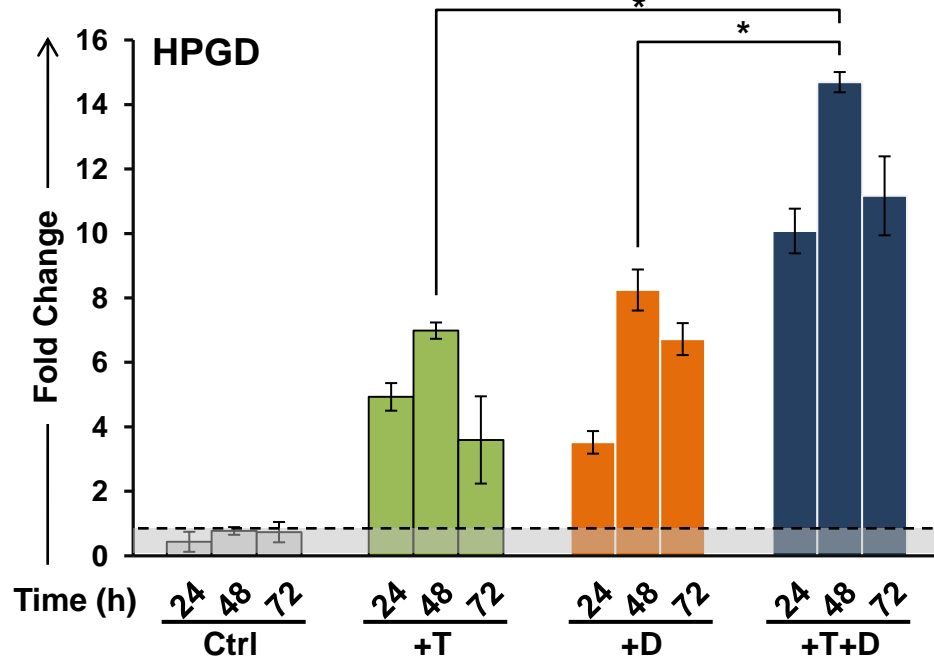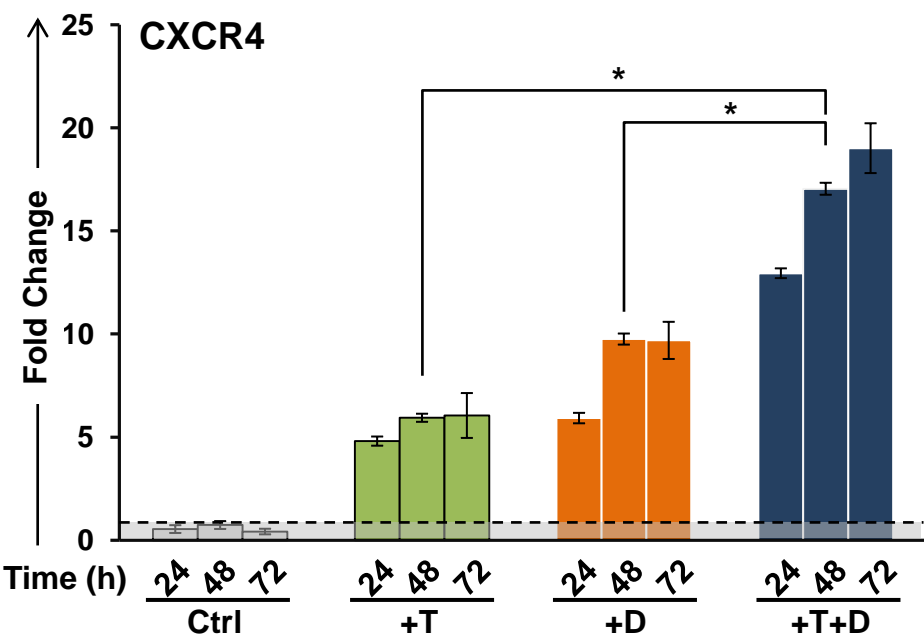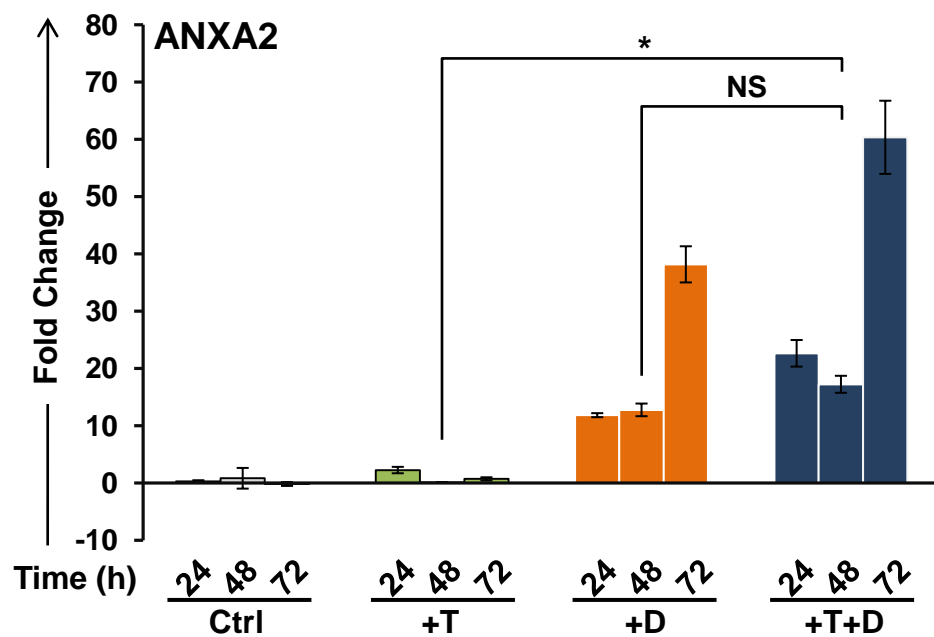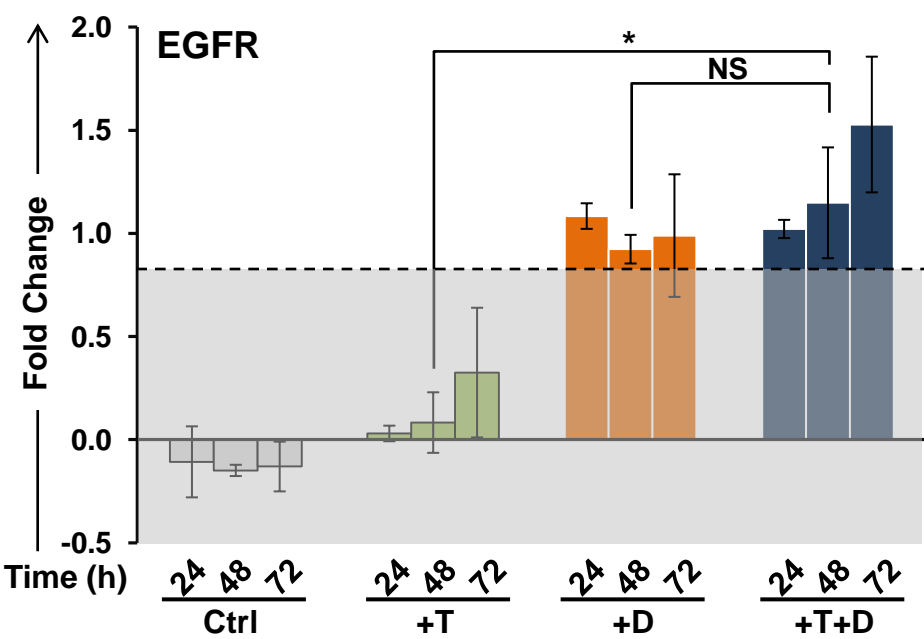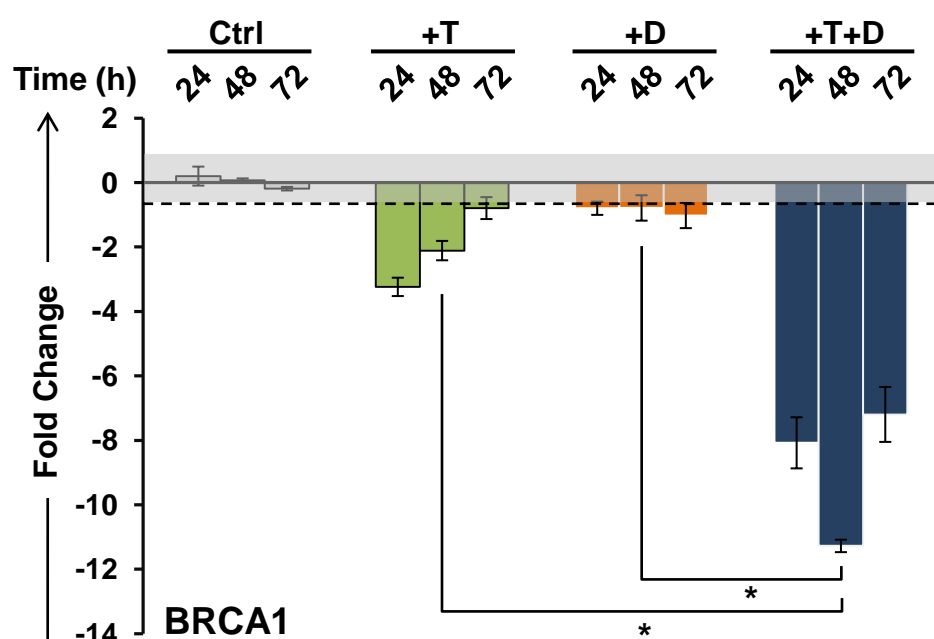

Supplement: Additional file 5 — Validation on changes in the mRNA levels of selected genes involved in lipid metabolism, angiogenesis, calcium induced signaling and DNA repair. CYP2U1, HPGD, CXCR4, ANXA2, EGFR and BRCA1 transcripts were measured over a 72 h time course in LNCaP cells after treatment with 5 nM T and 100 nM 1,25(OH)2D3. Fold changes greater than 0.8 are statistically significant; values within the shaded areas in each graph are not significantly modulated (0.8 or below after transformation). Comparisons between different treatment groups were analyzed using one-way ANOVA; differences were considered significant if p < 0.05 (*), NS: not significant. Significant changes (p < 0.05) in at least two out of three time points were required for the changes to be considered biologically relevant. Note: Scales on the ordinate axis vary from transcript to transcript. [file 1476-4598-10-58-S5.PDF]

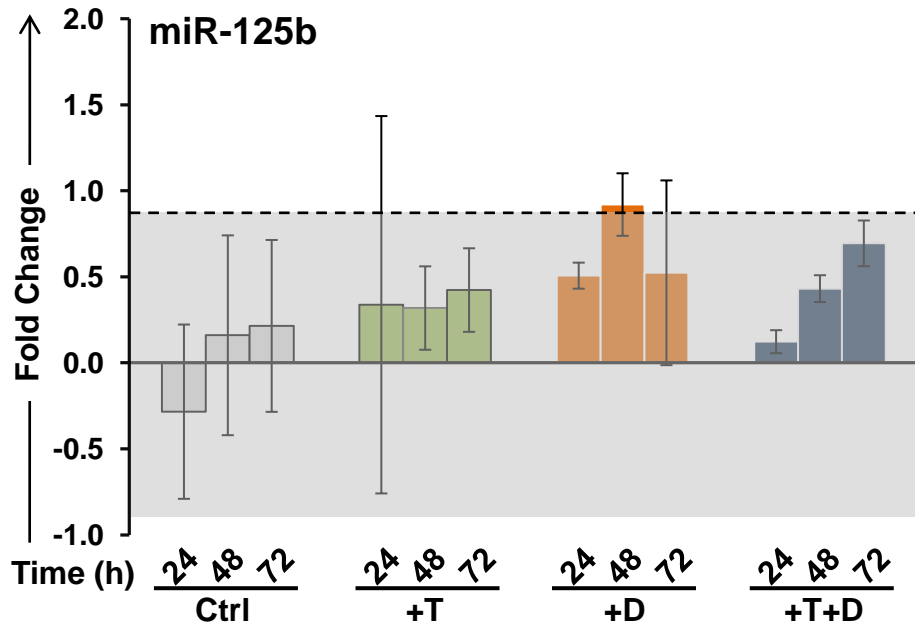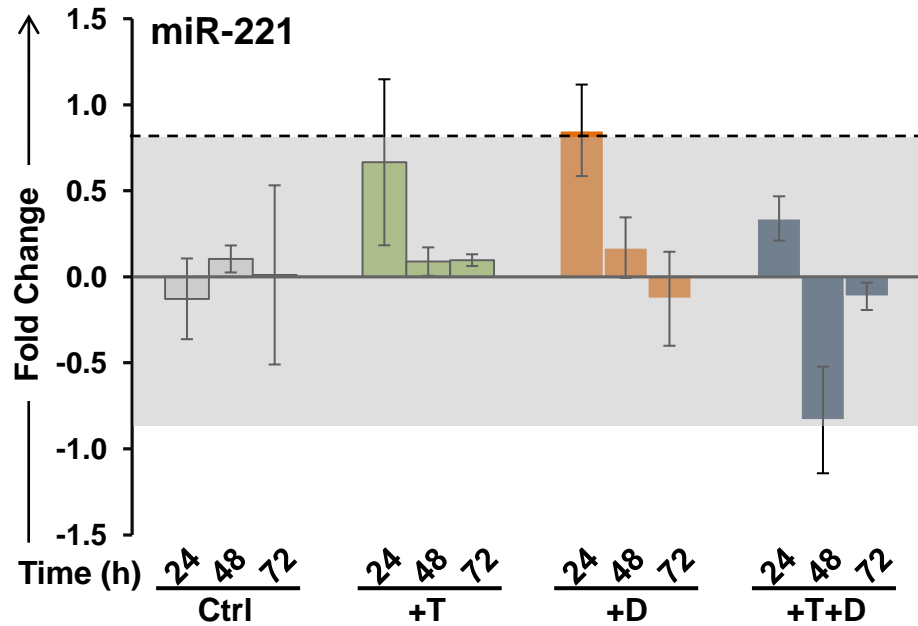

Supplement: Additional file 8 — Effects of T and 1,25(OH)2D3 on the expression of miR-125b and miR-221 in LNCaP cells. LNCaP cells were treated with 5 nM T and 100 nM 1,25(OH)2D3 alone and in combination and transcript levels were measured by TaqMan® qPCR over a 72 h time course. Other details as shown in Figure S4. [file 1476-4598-10-58-S8.PDF]
